# The European Register of Cystic Echinococcosis, ERCE: state-of-the-art five years after its launch

**DOI:** 10.1186/s13071-020-04101-6

**Published:** 2020-05-07

**Authors:** Patrizia Rossi, Francesca Tamarozzi, Fabio Galati, Okan Akhan, Carmen Michaela Cretu, Kamenna Vutova, Mar Siles-Lucas, Enrico Brunetti, Adriano Casulli, the ERCE network, A. Angheben, M. Belhassen Garcia, N. Bagmet, S. Borys, S. Bresson-Hadni, F. Demonmerot, L. Millon, F. Bruschi, G. Calleri, C. Chemla, B. Castiglioni, L. G. Chianura, B. Dezsényi, M. F. Harandi, S. Nasibi, G. Ismailova, M. T. Giordani, V. Gjoni, R. Shkjezi, L. Gogichaishvili, D. Goletti, F. Karim, E Lapini, S. Mastrandrea, F. Lötsch, G. Menozzi, R. Corsini, P. Milhailescu, S. Orsten, A. Paugam, M. Ramharter, A. Recordare, F Salvador, A. Teggi, C. Torti, G. Vitale, A. Vola, M. Mariconti, R. Lissandrin, M. Wallon, L. Zammarchi, F. Bartalesi

**Affiliations:** 1 European Reference Laboratory for Parasites, Department of Infectious Diseases, Istituto Superiore di Sanità, Rome, Italy; 2 WHO Collaborating Centre for the Epidemiology, Detection and Control of Cystic and Alveolar Echinococcosis, Department of Infectious Diseases, Istituto Superiore di Sanità, Rome, Italy; 3 DG-INF - Information Technology Service, Istituto Superiore di Sanità, Rome, Italy; 4 Department of Radiology, Faculty of Medicine, Hacettepe University, Ankara, Turkey; 5 C. Davila University of Medicine and Pharmacy, Colentina Clinical Hospital, Bucharest, Romania; 6 Specialised Hospital of Infectious and Parasitic Diseases “Prof. Ivan Kirov”, Department of Infectious, Parasitic and Tropical Diseases, Medical University, Sofia, Bulgaria; 7 Instituto de Recursos Naturales y Agrobiología de Salamanca, CSIC, Salamanca, Spain; 8 Department of Clinical Surgical Diagnostic and Paediatric Sciences, University of Pavia, Pavia, Italy; 9 Division of Tropical and Infectious Diseases, San Matteo Hospital Foundation, Pavia, Italy

**Keywords:** Cystic echinococcosis, Register, Public health awareness, Case series

## Abstract

**Background:**

The real burden of human cystic echinococcosis (CE) remains elusive, due to the peculiar characteristics of the disease and the heterogeneous and incomplete data recording of clinical cases. Furthermore, official notification systems do not collect pivotal clinical information, which would allow the comparison of different treatment outcomes, and thus circumvent the difficulty of implementing clinical trials for CE. The Italian Register of CE (RIEC) was launched in 2012 and expanded in 2014 into the European Register of CE (ERCE). The primary aim of the ERCE was to highlight the magnitude of CE underreporting, through the recording of cases that were not captured by official records. We present an overview of data collated in the ERCE and discuss its future, five years after its inception.

**Methods:**

The ERCE database was explored on March 31st 2019; data concerning participating centres and registered cases were descriptively analysed.

**Results:**

Forty-four centres from 15 countries (7 non-European) were affiliated to the ERCE. Thirty-four centres (77%) registered at least one patient; of these, 18 (53%) recorded at least one visit within the past 18 months. A total of 2097 patients were registered, 19.9% of whom were immigrants. Cyst characteristics were reported for at least one cyst at least in one visit in 1643 (78.3%) patients, and cyst staging was used by 27 centres. In total, 3386 cysts were recorded at first registration; mostly located in the liver (75.5%). Data concerning clinical management could be analysed for 920 “cyst stage-location-management” observations, showing great heterogeneity in the implementation of the stage-specific management approach recommended by the WHO.

**Conclusions:**

The ERCE achieved its goal in showing that CE is a relevant but neglected public health problem in Europe and beyond, since a proportion of patients reaching medical attention are not captured by official notification systems. The ERCE may provide a valuable starting platform to complement hospital-derived data, to obtain a better picture of the epidemiology of clinical CE, and to collect clinical data for the issue of evidence-based recommendations. The ERCE will be expanded into the International Register of CE (IRCE) and restructured aiming to overcome its current criticalities and fulfil these aims.
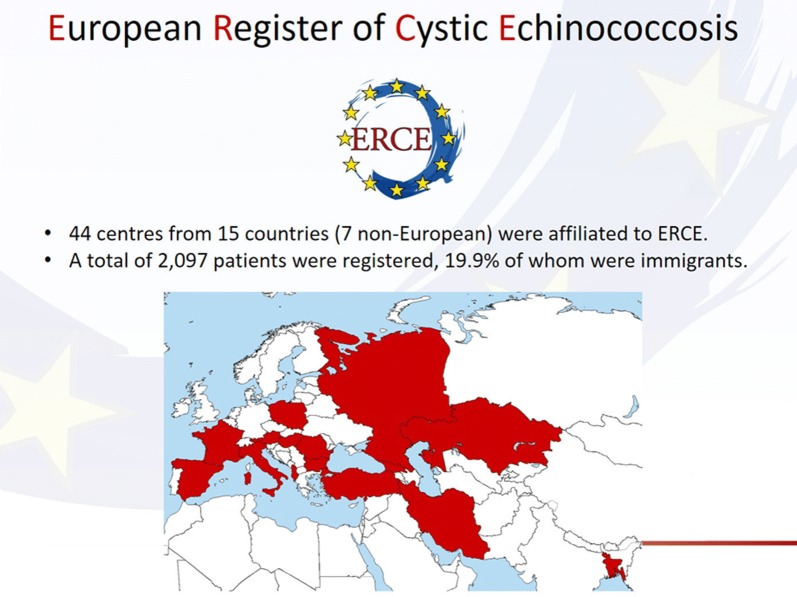

## Background

Cystic echinococcosis (CE) is a parasitic zoonosis, listed among the neglected diseases for which the World Health Organization (WHO) advocates concentrated control efforts [1]. Human CE is caused by the tapeworm *Echinococcus granulosus* (*sensu lato*) species complex, naturally transmitted between canids (mainly the domestic dog) and ungulates (mainly livestock, especially sheep) in a faecal-oral and predator-prey cycle. After the accidental ingestion of infective eggs by humans, the parasite develops as fluid-filled cysts (metacestodes) in organs and tissues, mainly the liver and lungs [2]. CE is mostly endemic in rural areas of China, Central Asia, South America, the Mediterranean, East Africa, and Australia, where livestock breeding is practiced [3].

Current CE burden estimates indicate globally about 300,000 disability adjusted life years, and an annual cost of about 200 million USD for human treatment of CE [4]. However, these estimates, based on highly heterogeneous and incomplete data sources, are largely underestimated, resulting in misperception of the magnitude of CE public health impact. Figures actually triplicate when accounting for estimated underreporting of cases reaching medical attention, not taking into consideration the disease burden carried by infected people not accessing health care [4]. Underreporting at national and supra-national level (e.g. to the European Centre for Diseases Prevention and Control, ECDC) derives from many factors. In Europe, CE notification requirements vary and are applied differently from country to country [5]. Further, the origin and type of available official data is heterogeneous (case-based or aggregated, from laboratories or hospitals or physicians), derived mainly from hospitalization records [5–8]. Patients managed as outpatients are largely not captured by official statistics, and notification of hospital cases to a central level is often incomplete. Moreover, in official European records, species differentiation between *E. granulosus* (*s.l.*) and *Echinococcus multilocularis*, the agent of alveolar echinococcosis (AE), is limited, adding to the inaccuracy of data [9, 10].

Official data are collected at national and supra-national level for epidemiological purposes. Pivotal data characterizing the course and clinical decision making for the management of this complex infection, such as cyst stage, clinical management details, and outcomes, are not collected. CE is a chronic, generally low-prevalence disease with heterogeneous clinical manifestations (including cyst number, location, size, stage, symptoms, complications and so on) that develops over years and requires a very long follow-up period to ascertain the outcome of the chosen clinical management approach [11–13]. Consequently, prospective clinical trials are virtually impossible to perform and treatment recommendations are largely based on expert opinion, which are, in turn, not widely followed [14, 15]. This often results in the administration of inappropriate treatments, which burden both patients and health care systems [16, 17].

In 2012, the WHO Collaborating Centre for Clinical Management of Cystic Echinococcosis (San Matteo Hospital Foundation, University of Pavia, Italy) and the Italian National Health Institute (Istituto Superiore di Sanità, Rome, Italy), through funding from the Sardinian Experimental Zooprophylactic Institute (Sassari, Italy), launched the Italian Register of Cystic Echinococcosis (RIEC) [18]. This aimed to respond to a long-standing need for an accessible register of CE cases, built to consider the peculiar characteristics of this infection. In 2014, RIEC was restructured and expanded into the European Register of Cystic Echinococcosis (ERCE), in the context of the European FP7 project “HERACLES” [19]. The main aims of the register were: (i) to indicate the magnitude of the problem represented by CE (mainly by recording cases otherwise not captured by official records, such as those only managed as outpatients); (ii) to bring the importance of CE to the attention of health authorities; (iii) to encourage public health policies for its control; and (iv) to support epidemiological, biological, and clinical research on CE by establishing a prospective case retrieval through the collection of data in a harmonized manner. Moreover, the ERCE supports the biobank repository of biological material derived from patients with CE and *E. granulosus* (*s.l.*) parasites (EchinoBioBank), established in Salamanca, Spain, in the context of the HERACLES project.

The structure and requirements of the ERCE are shown in Table 1 and have been described in detail in a previous publication on the first meeting of the ERCE network that took place in Rome in 2015 [19]. Here, we present an overview of data present in the ERCE and discuss its future development, five years after its launch.

**Table 1:** Schematic overview of the structure and features of the ERCE

Item	Feature
Patients enrolled in ERCE	With confirmed or probable CE (according to WHO-IWGE 2010 Expert Consensus definition) In- and out-patients All ages and both sexes Diagnosed at the time of the recorded visit or previously; follow-up visits
Data recorded for each patient	Personal data: year of first diagnosis of CE Clinical data: cyst(s) localization, size and stage History of treatments and treatment/management approach currently being received Biological samples collected (if any)
ERCE structure	Multicentre database located within the secured IT network of the Italian National Institute of Health (Istituto Superiore di Sanità, ISS) in Rome Currently available in English, Italian, Romanian, Bulgarian and Turkish Organized in sheets where patient data are recorded Each registered patient is automatically given a unique ERCE ID code Data are uncoupled and pseudonymized Only the physician who entered the patient’s data and the ERCE manager can access the record
ERCE users	Physicians working in health centres where patients with CE are managed Join the ERCE network voluntarily Are provided with personal credentials to login into the register Different roles are envisaged: - the “person in charge” in each centre enters patients’ data - the “supervisor” in each centre can read only data of his/her centre - the Register “coordinator” has access to and can download data from all national centres Possibility to have a National Centre coordinating data collection from centres of the Country^a^
Requirements to join ERCE	To be a physician working in centres where CE patients (in- and out-patients) are visited To obtain the approval from the ethics committee of each centre/country involved (although the implementation of the Register is only observational and does not involve clinical experimentation)
Ownership of data	Data from individual centres belong to the individual centres themselves The coordinator can only use cumulative data for periodic presentations on the progress of the ERCE Publication of data requires the consent of the individual centres
Confidentiality and security	ERCE was approved by the ISS ethics committee (Prot. PRE-C-915/14 of November 25th, 2014), extending the agreement to the Italian Register of CE (Ns Prot. CE/12/347 of May 7th, 2012) ERCE complies with EU Regulation on the protection and use of personal data (Reg. EU 2016/679) Two informed consent forms must be signed by patients at initial registration to allow: - their data to be recorded in the Register - their biological samples to be shipped to the Echino-Biobank The ISS datacentre, through the Azure Backup Server System, makes a complete backup every night and an online backup copy on cloud daily, weekly, monthly and yearly, stored for up to 20 years

^a^France and Iran

## Methods

The complete ERCE database explored on March 31st 2019. Analysed data referred to participating centres (number, country, date of joining the ERCE network, centre’s activity identified as recording of visits in the previous 18 months, total recorded cases and follow-up visits), and registered cases (sex, age, country of birth, cysts characteristics and clinical management at each visit). For the analysis of clinical management, only the data of patients whose records included cyst stage, location, and clear indication of management at defined visits, occurring after the date of first recording in ERCE, were extrapolated from the database. The descriptive analysis of the stage-specific management of CE was carried out as if each observation of the set “cyst stage-location-management” was an independent observation. That is, an individual cyst in a given localization in a patient, which was observed N times in different cyst stage and/or assigned to a different clinical management option, was analysed here as “N observations”. Only when a change in cyst stage and/or management allocation was recorded, a new observation was scored. Data were summarized as counts and percentages.

## Results

**Fig. 1:**
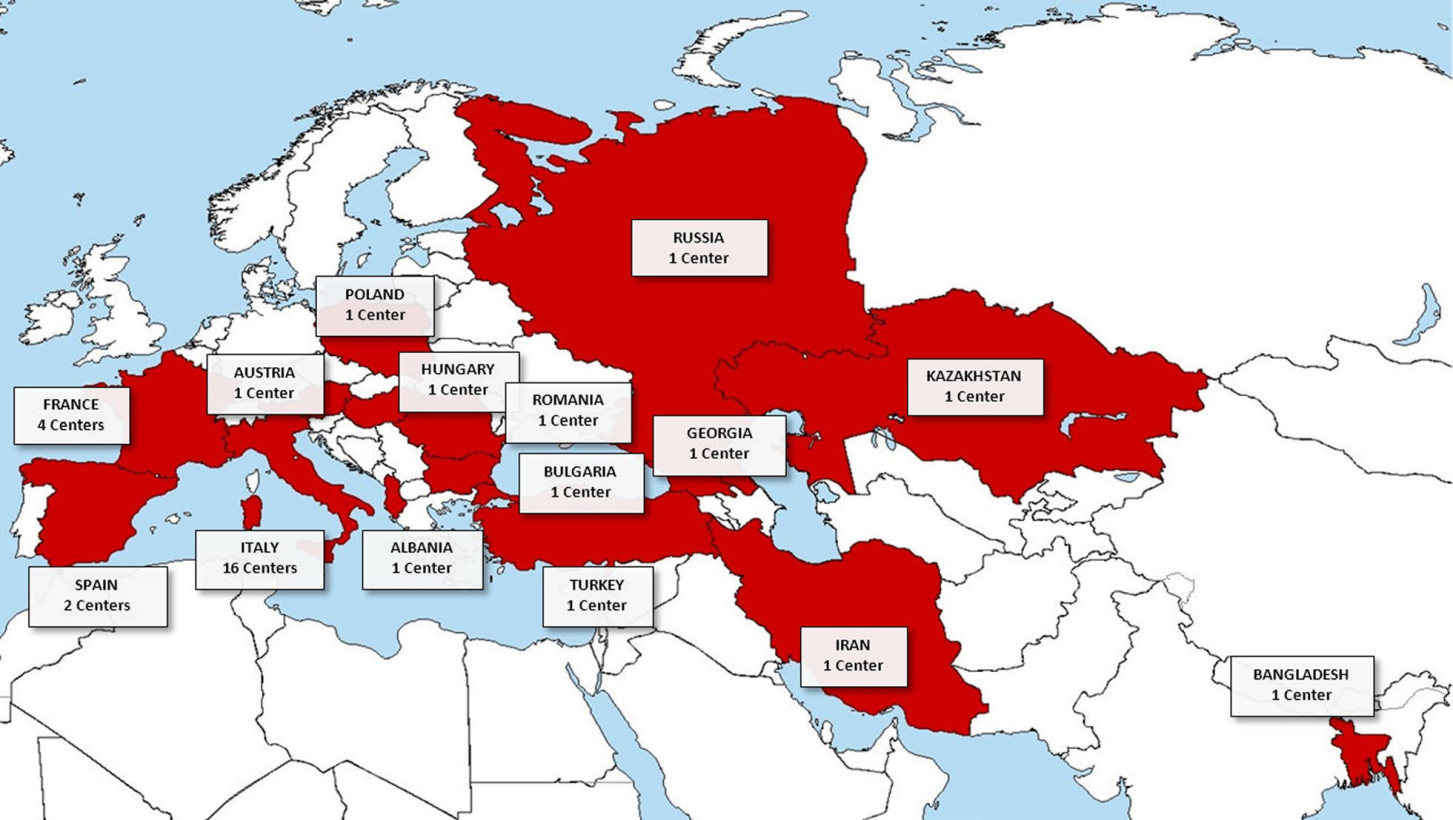
Countries and number of centres involved in the ERCE. Only the centres that registered at least one patient are included

**Fig. 2:**
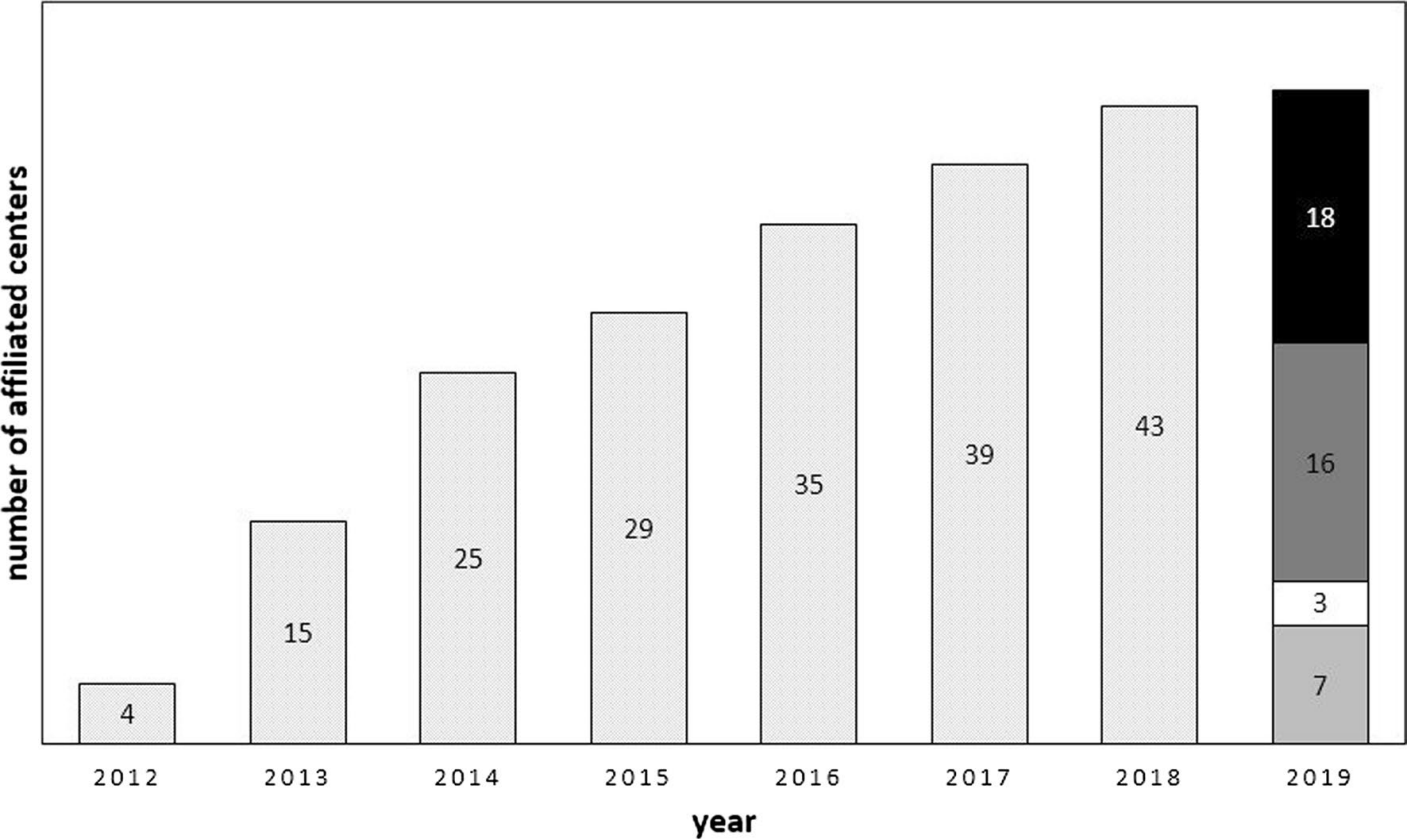
Affiliation and current activity of the ERCE network centres. Bars represent the cumulative number of affiliated centres at each year of activity of the RIEC (2012–2013) and ERCE (2014 onwards). Current activity status of affiliated centres is shown in the 2019 bar. Light grey indicates centres that never entered patients’ information in the database; white indicates 3 centres in France that send their data to a National reference centre which entered these patients’ information in the database; dark grey indicates centres that entered at least 1 patients’ information in the database since their affiliation but did not enter new data in the past 18 months; black indicates centres that entered new patients’ information in the database in the past 18 months

On March 31st 2019, 44 centres from 15 countries (Fig. 1), seven of which are non-European, were affiliated to ERCE. Centres joining the ERCE steadily increased over the years (Fig. 2). Of the affiliated centres, 34 (77%) registered at least one patient and, of these, 18 (53%) recorded at least one visit within the past 18 months (Figs. 1, 2). The absence of data from those centres that never entered patients’ information in the database (Fig. 2) is most likely due to a variable combination of reasons. These include absence of new diagnoses of CE or follow-up visits of patients managed in the centre (especially in hospitals that are not referral centres for CE or that joined the register only very recently), duties overload or change of position of the clinician who originally joined voluntarily the network.

**Fig. 3:**
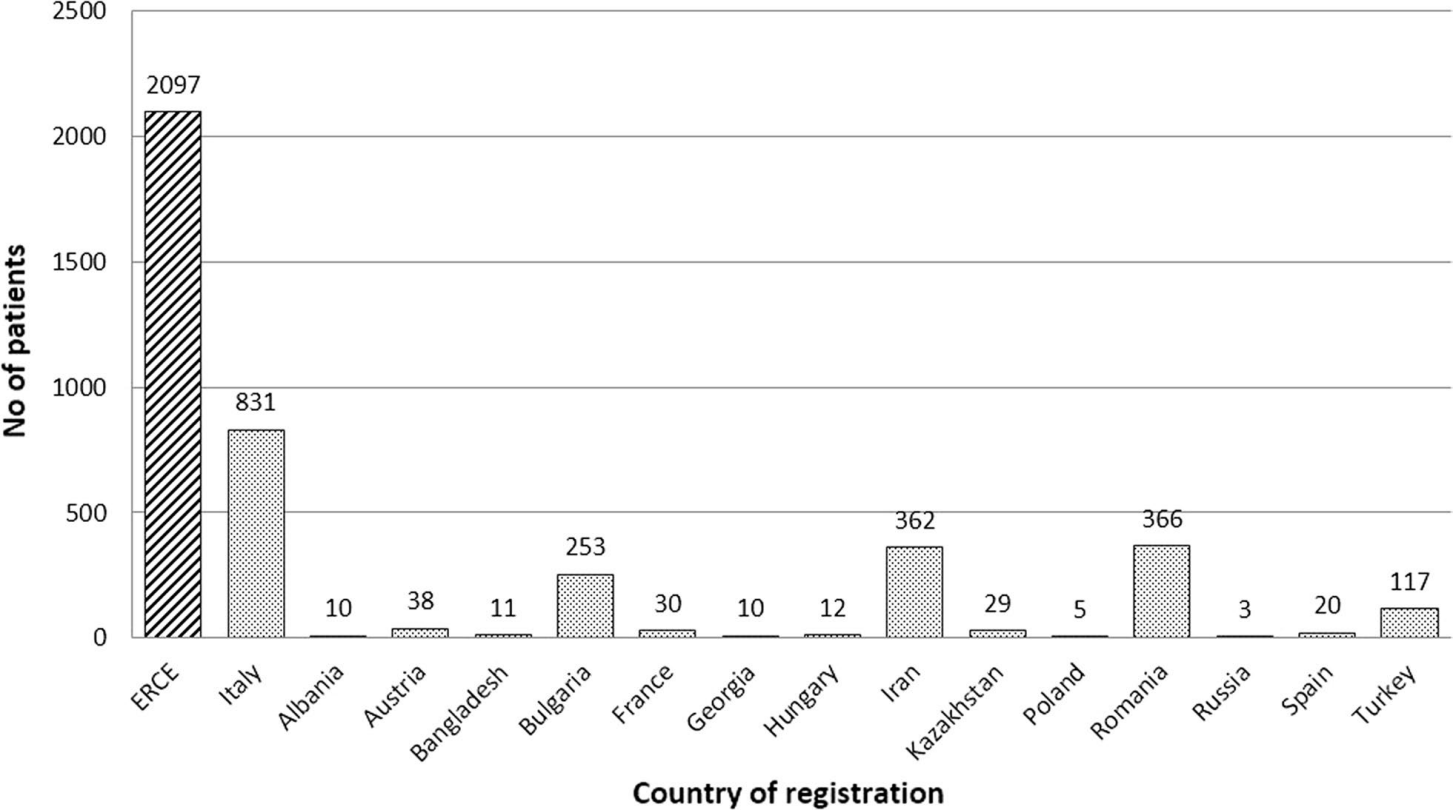
Number of patients in the ERCE by country of registration

**Fig. 4:**
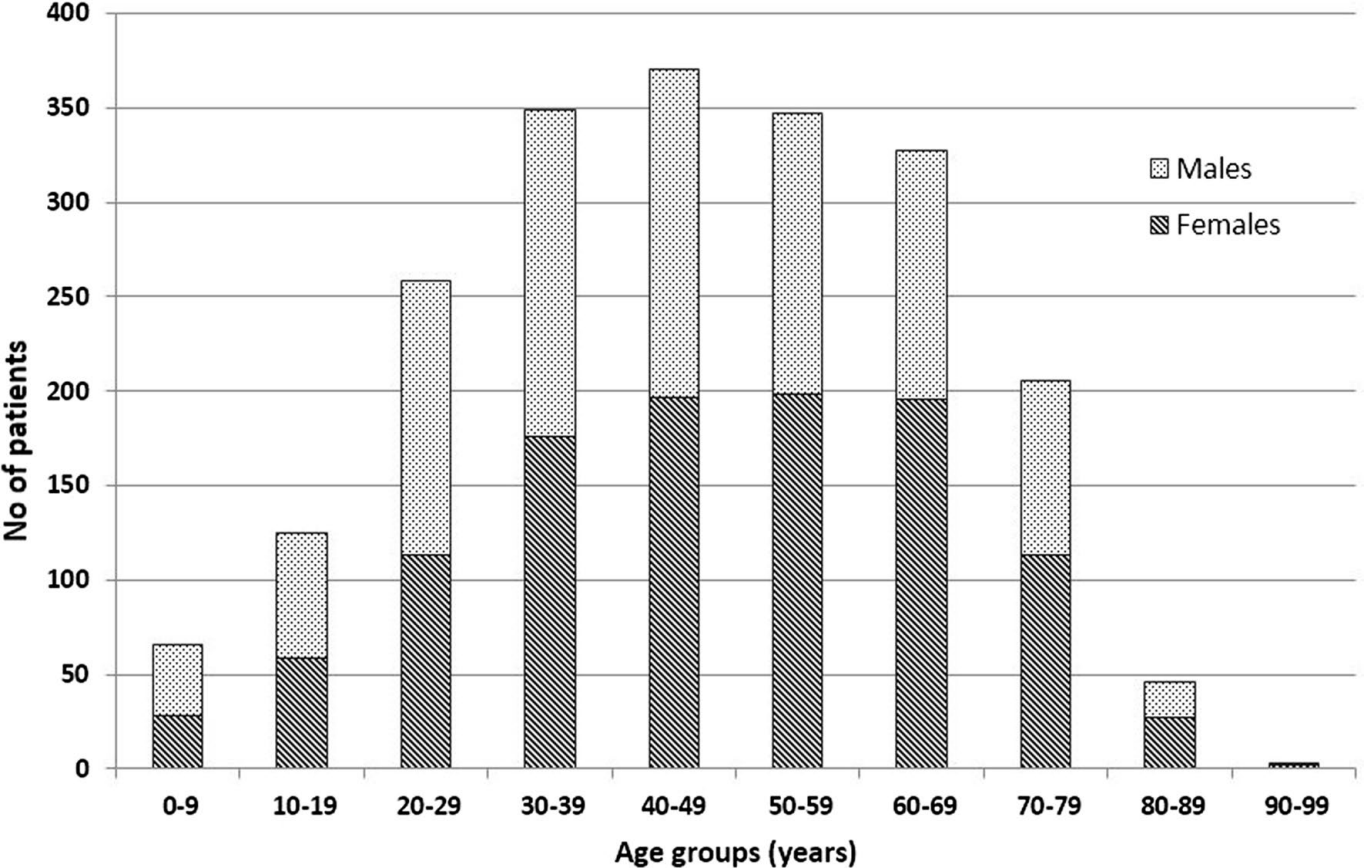
Age groups and sex of patients registered in the ERCE

A total of 2097 patients were registered at the date of data extraction; of these, 831 (40%) were registered in 16 Italian centres (Fig. 3). Notably, a total of 119 patients (5.7% of all patients registered in ERCE) were detected during the ultrasound-based population screening carried out in 2014–2015 in Bulgaria, Romania and Turkey, in the context of the HERACLES project [20]. These patients are 12.2% of those registered in Sofia (Bulgaria), 9.6% of those registered in Bucharest (Romania), and 45.3% of those registered in Ankara (Turkey). The distribution of patients by sex and age at first registration in the ERCE is shown in Fig. 4; 53.1% of patients were females and 46.9% males, showing this even distribution between sexes throughout all age groups, most patients (66.4%) were in the adult 30–69 years age group (mean age 46.20 years, range 2–97 years). Additional file 1: Table S1 shows the country of birth of registered patients. Immigrants (defined as patients with CE who were born in a country different to that of enrolment), constituted 19.9% of the total registered patients; however, their relative percentage by centre, unsurprisingly, varied greatly among centres.

Echinococcal cyst characteristics (organ involved and/or cyst stage) were reported by 28 centres (representing 82.4% of centres having registered at least one patient) at least in one visit for at least one cyst in 1643 (78.3%) of patients. Although cyst staging according to the WHO-IWGE (Informal Working Group on Echinococcosis) classification [14] was used by 27 centres (i.e. 79.4% of centres having registered at least one patient); however, cyst stage, when applicable, was not constantly reported in all patients registered in individual centres and/or at each visit of the same patient. At first registration, for example, cyst staging was not reported for one-third of registered cysts (Table 2).

**Table 2:** Cyst characteristics at first registration in the ERCE (for the subset of patients for whom these data were recorded by the data entering clinician)

No. of cysts per patient^a^ (*N* = 1966)	No. of patients (%)	WHO-IWGE stage	Liver right lobe (%)	Liver left lobe (%)	Liver both lobes (%)	Lung right (%)	Lung left (%)	Lung both (%)	Other localizations (%)	Total (%)
1	1162 (59.1)	Not specified	342 (16.9)	115 (23.9)	26 (47.3)	279 (86.9)	224 (89.6)	21 (80.8)	77 (30.1)	1084 (31.8)
2	380 (19.3)	CE1	325 (16.1)	65 (13.5)	3 (5.4)	13 (4.0)	8 (3.2)	2 (7.7)	48 (18.8)	464 (13.6)
3	136 (6.9)	CE2	190 (9.4)	44 (9.1)	5 (9.1)	5 (1.6)	4 (1.6)	0	24 (9.4)	272 (8.0)
4	48 (2.4)	CE3a	133 (6.6)	30 (6.2)	3 (5.4)	6 (1.9)	3 (1.2)	0	6 (2.3)	181 (5.3)
5	23 (1.2)	CE3b	199 (9.8)	45 (9.4)	6 (10.9)	2 (0.6)	0	0	31 (12.1)	283 (8.3)
6	10 (0.5)	CE4	478 (23.6)	105 (21.8)	3 (5.4)	8 (2.5)	6 (2.4)	2 (7.7)	39 (15.3)	641 (18.8)
7	7 (0.4)	CE5	201 (9.9)	31 (6.5)	0	4 (1.2)	2 (0.8)	0	18 (7.1)	256 (7.5)
8	1									
9	1									
≥ 10	17 (0.9)	CL	33 (1.6)	15 (3.1)	3 (5.4)	1 (0.3)	0	1 (3.8)	6 (2.3)	59 (1.7)
CL	27 (1.4)	Post-surgery	120 (5.9)	31 (6.5)	6 (10.9)	3 (0.9)	3 (1.2)	0	6 (2.3)	169 (5.0)
Post-surgery	153 (7.8)	Total	2021 (59.7)	481 (14.2)	55 (1.6)	321 (9.5)	250 (7.4)	26 (0.8)	255 (7.5)	3409

^a^At enrolment

*Abbreviations*: CL, cystic lesion (suspect cyst according to the WHO-IWGE)

At first registration in ERCE, the majority (*n* = 1162/1966; 59.1%) of patients who had information on cyst number recorded had a single cyst; 153 (7.8%) had no cysts and were registered as patients with only post-surgical cavities/scars (Table 2). In total, 3409 cysts were recorded at first registration; the most frequent localization was the liver (*n* = 2557 cysts; 75.5%), followed by the lungs (*n* = 597 cysts; 17.5%) (Table 2). Other localizations, accounting for 7.5% of recorded cysts, included abdominal cavity, bone, central nervous system, heart, kidneys, muscle, pancreas, pelvis, skin and subcutaneous tissue, and spleen; 78 (3.9%) patients had cysts in more than one organ. Table 2 also summarizes the distribution of cyst stages recorded at first registration, classified according to the WHO-IWGE classification [14].

Treatments carried out prior to ERCE registration, as well as clinical management upon registration and subsequent follow-up visits, are also recorded in the register. Seventeen centres (representing 50.0% of centres having registered at least one patient) recorded at least once one follow-up visit for at least one patient; in these centres, 435 patients had at least one follow-up visit recorded (a median of 21% patients per centre, ranging from 1.6% to 84.2%). Data concerning the clinical management of patients could be analysed for 523 patients registered in 24 centres, for 726 cysts. As 82 patients had at least one follow-up visit recorded in which a change in cyst stage and/or management allocation was indicated, the total number of “cyst stage-location-management” observations analysed was 920 (Additional file 1: Table S2). A stage-specific approach is recommended by the WHO-IWGE for asymptomatic CE cysts of the liver. We analysed the data concerning the clinical approach of hepatic cysts according to cyst stage. Results are summarized in Table 3. These results need to be evaluated while acknowledging the fact that no information is recoded in the ERCE register concerning symptoms or other clinical factors that may induce the treating physician to deviate from the recommended stage-specific approach. The clinical management used for cysts in other organs is summarized in Additional file 1: Table S2. One centre from a non-European country, systematically did not indicate any cyst stage and all cases were managed surgically, with no indication of associated albendazole prophylaxis in the virtual totality of cases.

**Table 3:** Clinical management approach of hepatic CE cysts by stage

Stage	*N*^a^	ABZ	Surgery with no specification of prophylaxis with ABZ	Surgery with specified associated prophylaxis with ABZ	Percutaneous treatment with no specification of prophylaxis with ABZ	Percutaneous treatment with specified associated prophylaxis with ABZ	Watch-and-wait
CE1	159	66	35	19	12	25	2
CE2	100	41	22	21	0	15^b^	1
CE3a	94	41	5	6	2	11	29
CE3b	210	83	4	60	0	4	59
CE4-CE5	210	17^c^	2	14	0	0	177

^a^Number of “cyst stage-location-management” observations

^b^11 cysts in the CE2 stage were treated by percutaneous treatment plus ABZ in the Turkish center where the non-PAIR percutaneous technique is performed, as envisaged by the WHO-IWGE expert consensus

^c^For 6 CE4 cysts, ABZ treatment was applied due to the concomitant presence of other CE cyst stages

*Abbreviation*: ABZ, albendazole

## Discussion

The primary aim of the ERCE, at its launch, was prominently of a public health perspective, to indicate to stakeholders the magnitude of the problem represented by CE and its underreporting, through the recording of cases not captured by official records [19]. Since its launch, new centres are joining the ERCE over time, demonstrating that CE, although neglected, is of interest for clinicians diagnosing and managing patients with CE, in European and non-European countries alike. In the time between data analysis and the writing of this paper, a 45th centre, in Afghanistan, joined the ERCE. This is of particular interest because no epidemiological data on CE are available in this country, published data being limited to case reports of patients from Afghanistan diagnosed in other countries [3]. In our cohort described here, 39 patients from Afghanistan were recorded in Iran and Austria ERCE-affiliated centres (Additional file 1: Table S1). Data collected in the ERCE, therefore, could highlight the neglected status of CE in endemic countries and constitute an important starting point to tackle this problem.

Since joining the register and recording of patients is voluntary and relies entirely on the time and goodwill of single clinicians, it is difficult to compare the figures recorded in the ERCE with those of official records. However, data collected in the ERCE show that a proportion of patients reaching medical attention are indeed managed with approaches not requiring hospitalization, and are therefore largely not captured by those notification systems which are based only on hospital discharge records [21]. We found, unsurprisingly, that a variable proportion of patients originate from countries different from the country of registration, both within and outside Europe. The increase of the migration phenomenon at global level [22] seems to cause an increase in CE cases seen in non-endemic areas [23] as well as an additional burden in CE-endemic countries. The ERCE could be, therefore, a useful tool to complement hospital-derived data, to obtain a better picture of the epidemiological situation of clinical CE and support public health analyses and planning of targeted interventions. Furthermore, the ERCE aims to capture only probable or confirmed CE cases, based on the current definition of the WHO-IWGE [14], with diagnosis based on the visualization on imaging of a lesion with features compatible with or pathognomonic of CE. This is different from the case definition of “echinococcosis” adopted at national and supra-national level in Europe, as currently official data not only do not distinguish between cases of CE and AE, but also allow patients positive solely on serology but with no evidence of actual cysts, to be recorded as “confirmed echinococcosis” cases [10].

In contrast to official data reports, the ERCE has been structured to collect prospectively and in a harmonized manner several clinical features of CE cases over follow-up visits. CE is a generally low-prevalence, chronic infection with extremely variable clinical presentations; it cannot be managed using a “one-fits-all” approach, and years-long follow-up is required to ascertain the outcome of any clinical management approach. As a result, prospective clinical trials are extremely difficult to conduct. The WHO-IWGE encouraged the clinical community to join shared data platforms to collect prospectively and analyse highly standardized clinical data as, in such a situation, it is possible to obtain valid comparisons of different treatments using data from observational studies, provided specific prerequisites are fulfilled [24]. The ERCE can thus be a valuable template from which further tailoring a tool fulfilling these prerequisites, to accomplish this ambitious goal.

At the time of the present analysis, it was possible to evaluate the matching cyst characteristics-management option only for about 25% of patients. On the one hand, this highlights the problem of suboptimal data completeness and quality, which derives from the voluntary nature of adherence to and feeding data into the register. On the other hand, this result may also have its roots in the scarce knowledge and application of the CE cyst staging system and the stage-specific clinical management approach recommended by the WHO-IWGE [13, 14]. This is exemplified by the case of one centre in a non-European country, where, systematically, no cyst stage was ever indicated, and all cases were reported to have been managed surgically. Although many variables, other than cyst stage, that may influence a physician to choose a treatment option, are not currently captured in the ERCE, where cyst staging is being used at least some stage-specific approaches seem to be applied. For example, the “watch and wait” approach was reported for over 80% of the observations concerning hepatic inactive (CE4-CE5) cysts. It is plausible to deduce that knowledge of cyst staging and applying the stage-specific approach are mutually related.

The ERCE, as it stands, suffers from several critical issues, especially regarding data quality and completeness, which will be addressed in the future, to fulfil the aims envisioned above. As mentioned, the voluntary nature of joining the register, and the reliance on the motivation of single clinicians to enter data for a period of long time, condition the variable quality of the entered data. However, additional informatics tools may be introduced to foster the accuracy and completeness of data entered, such as automatic crosschecking of data for incongruences. In addition, the option to upload cyst images should be implemented, to validate data before comparisons of different treatments is attempted. Finally, other prognostic factors relevant to treatment outcomes must be included, to allow drawing evidences from such case series.

## Conclusions

To conclude, the ERCE achieved its goal in showing that CE is a neglected but relevant public health problem in Europe and beyond, as indicated by the data collected so far and by the growing interest shown by European and non-European clinicians. Current data collected in the ERCE highlight the need for the development and implementation of a better notification system, at least at the European level, without which a realistic picture of the prevalence and burden of human CE cannot be achieved. In addition, the ERCE appears a valuable starting platform to draw future evidence-based recommendations, overcoming the virtual impossibility to perform clinical trials on CE [24]. The need to enter CE cyst stages and clinical management decisions may also constitute a vehicle of knowledge of the WHO-IWGE staging and recommendations among the clinician’s community.

Learning from the past five years, in the near future the ERCE will be expanded to the online International Register of CE (IRCE) within the framework of the “PERITAS” project funded by the European platform EU-LAC Health (http://eulachealth.eu/) and the National funding agencies of the participating institutions. The ERCE will be restructured into the IRCE aiming to answer relevant questions regarding the clinical management of CE.

## Supplementary information

**Additional file 1: Table S1.** Country of birth for patients registered in ERCE centres. **Table S2.** Clinical management of cysts by stage and location.

## Data Availability

The datasets generated and analysed during the current study are not publicly available for personal data protection, in compliance with the Regulation (EU) 2016/679 and, formerly, with the Directive 96/46/EC, but are available from ERCE coordinators in a pseudonymized manner, subject to formal and motivated request and consent from relevant individual centres. All data are collected only upon obtainment of patient’s written consent, each Centre participating to the ERCE network owing data of patients registered in its institution.

## Abbreviations

CE: cystic echinococcosis
ECDC: European Centre for Diseases Prevention and Control
ERCE: European Register of Cystic Echinococcosis
IRCE: International Register of Cystic Echinococcosis
RIEC: Registro Italiano dell’Echinococcosi Cistica (Italian Register of Cystic Echinococcosis)
WHO: World Health Organization
WHO-IWGE: World Health Organization - Informal Working Group on Echinococcosis
