# Supplementary material for: The European Register of Cystic Echinococcosis, ERCE: state-of-the-art five years after its launch
Source: Parasit Vectors. 2020 May 7;13:236. doi: 10.1186/s13071-020-04101-6 (PMC7206799; doi:10.1186/s13071-020-04101-6)
Supplement: Supplementary file 1 — Additional file 1: Table S1. Country of birth for patients registered in ERCE centres. Table S2. Clinical management of cysts by stage and location. [file 13071_2020_4101_MOESM1_ESM.docx]

**Additional file 1: Table S1.** Birth countries of patients registered in ERCE centers

| **Country of birth** | **Country of enrollment**  **Number of patients** | | | | | | | | | | | | | | | |
| --- | --- | --- | --- | --- | --- | --- | --- | --- | --- | --- | --- | --- | --- | --- | --- | --- |
|  | **Albania** | **Austria** | **Bangladesh** | **Bulgaria** | **France** | **Georgia** | **Hungary** | **Iran Islamic Rep** | **Italy** | **Kazakhstan** | **Poland** | **Romania** | **Russia** | **Spain** | **Turkey** | **Total** |
| **Albania** | **10** | **-** | **-** | **-** | **-** | **-** | **-** | **-** | **50** | **-** | **-** | **-** | **-** | **-** | **-** | **60** |
| **Algeria** | **-** | **-** | **-** | **-** | **8** | **-** | **-** | **-** | **5** | **-** | **-** | **-** | **-** | **-** | **-** | **13** |
| **Afghanistan** | **-** | **1** | **-** | **-** | **-** | **-** |  | **38** | **-** | **-** | **-** | **-** | **-** | **-** | **-** | **39** |
| **Austria** | **-** | **7** | **-** | **-** | **-** | **-** | **-** | **-** | **-** | **-** | **-** | **-** | **-** | **-** | **-** | **7** |
| **Bangladesh** | **-** | **-** | **10** | **-** | **-** | **-** | **-** | **-** | **1** | **-** | **-** | **-** | **-** | **-** | **-** | **11** |
| **B. Herzegovina** | **-** | **1** | **-** | **-** | **1** | **-** | **-** | **-** | **2** | **-** | **-** | **-** | **-** | **-** | **-** | **4** |
| **Bulgaria** | **-** | **2** | **1** | **253** | **-** | **-** | **-** | **-** | **5** | **-** | **-** | **-** | **-** | **-** | **-** | **261** |
| **China** | **-** | **-** | **-** | **-** | **-** | **-** | **-** | **-** | **1** | **-** | **-** | **-** | **-** | **-** | **-** | **1** |
| **Ecuador** | **-** | **-** | **-** | **-** | **-** | **-** | **-** | **-** | **1** | **-** | **-** | **-** | **-** | **-** | **-** | **1** |
| **Egypt** | **-** | **-** | **-** | **-** | **-** | **-** | **-** | **-** | **1** | **-** | **-** | **-** | **-** | **-** | **-** | **1** |
| **Eritrea** | **-** | **-** | **-** | **-** | **-** | **-** | **-** | **-** | **2** | **-** | **-** | **-** | **-** | **-** | **-** | **2** |
| **France** | **-** | **-** | **-** | **-** | **3** | **-** | **-** | **--** | **1** | **-** | **-** | **-** | **-** | **-** | **-** | **4** |
| **Georgia** | **-** | **-** | **-** | **-** | **-** | **10** | **-** |  | **1** | **-** | **-** | **-** | **-** | **-** | **-** | **11** |
| **Ghana** | **-** | **-** | **-** | **-** | **-** | **-** | **-** |  | **2** | **-** | **-** | **-** | **-** | **-** | **-** | **2** |
| **Hungary** | **-** | **-** | **-** | **-** | **-** | **-** | **11** | **-** | **-** | **-** | **-** | **-** | **-** | **-** | **-** | **11** |
| **India** | **-** | **1** | **-** | **-** | **-** | **-** | **-** | **-** | **-** | **-** | **-** | **-** | **-** | **-** | **-** | **1** |
| **Iran I. R.** | **-** | **-** | **-** | **-** | **-** | **-** | **-** | **324** | **-** | **-** | **-** | **-** | **-** | **-** | **-** | **324** |
| **Iraq** | **-** | **1** | **-** | **-** | **-** | **-** | **-** | **-** | **-** | **-** | **-** | **-** | **-** | **-** | **-** | **1** |
| **Italy** | **-** | **-** | **-** | **-** | **-** | **-** | **-** | **-** | **513** | **-** | **-** | **-** | **-** |  | **-** | **513** |
| **Jordan** | **-** | **-** | **-** | **-** | **-** | **-** | **-** | **-** | **-** | **-** | **-** | **-** | **-** | **-** | **1** | **1** |
| **Kazakhstan** | **-** | **1** | **-** | **-** | **-** | **-** | **-** |  | **2** | **29** | **-** | **-** | **-** | **-** | **-** | **32** |
| **Lebanon** | **-** | **-** | **-** | **-** | **-** | **-** | **-** |  | **3** | **-** | **-** | **-** | **-** | **-** | **-** | **3** |
| **Libyan AJ** | **-** | **-** | **-** | **-** | **-** | **-** | **-** |  | **1** | **-** | **-** | **-** | **-** | **-** | **-** | **1** |
| **Macedonia** | **-** | **2** | **-** | **-** | **-** | **-** | **1** |  | **11** | **-** | **-** | **-** | **-** | **-** | **-** | **14** |
| **Morocco** | **-** | **-** | **-** | **-** | **6** | **-** | **-** |  | **88** | **-** | **-** | **-** | **-** | **-** | **-** | **94** |
| **Moldova** | **-** | **-** | **-** | **-** | **1** | **-** | **-** |  | **13** | **-** | **-** | **-** | **-** | **-** | **-** | **14** |
| **Pakistan** | **-** | **-** | **-** | **-** | **-** | **-** | **-** |  | **2** | **-** | **-** | **-** | **-** | **-** | **-** | **2** |
| **Peru** | **-** | **-** | **-** | **-** | **-** | **-** | **-** |  | **15** | **-** | **-** | **-** | **-** | **-** | **-** | **15** |
| **Poland** | **-** | **-** | **-** | **-** | **-** | **-** | **-** |  | **1** | **-** | **5** | **-** | **-** | **-** | **-** | **6** |
| **Romania** | **-** | **4** | **-** | **-** | **1** | **-** | **-** |  | **68** | **-** | **-** | **366** | **-** | **-** | **-** | **439** |
| **Russia** | **-** | **-** | **-** | **-** | **-** | **-** | **-** |  | **-** | **-** | **-** | **-** | **3** | **-** | **-** | **3** |
| **S. Arabia** | **-** | **-** | **-** | **-** | **-** | **-** | **-** |  | **1** | **-** | **-** | **-** | **-** | **-** | **-** | **1** |
| **Senegal** | **-** | **-** | **-** | **-** | **-** | **-** | **-** |  | **1** | **-** | **-** | **-** | **-** | **-** | **-** | **1** |
| **Serbia** | **-** | **4** | **-** | **-** | **-** | **-** | **-** |  | **8** | **-** | **-** | **-** | **-** | **-** | **-** | **12** |
| **Slovenia** | **-** | **1** | **-** | **-** | **-** | **-** | **-** |  | **-** | **-** | **-** | **-** | **-** | **-** | **-** | **1** |
| **Spain** | **-** | **-** | **-** | **-** | **1** | **-** | **-** |  | **-** | **-** | **-** | **-** | **-** | **20** | **-** | **21** |
| **Syrian AR** | **-** | **-** | **-** | **-** | **2** | **-** | **-** |  |  | **-** | **-** | **-** | **-** | **-** | **-** | **2** |
| **Tunisia** | **-** | **-** | **-** | **-** | **1** | **-** | **-** |  | **16** | **-** | **-** | **-** | **-** | **-** | **-** | **17** |
| **Turkey** | **-** | **12** | **-** | **-** | **6** | **-** | **-** |  | **3** | **-** | **-** | **-** | **-** | **-** | **116** | **137** |
| **Ukraine** | **-** | **-** | **-** | **-** | **-** | **-** | **-** |  | **11** | **-** | **-** | **-** | **-** | **-** | **-** | **11** |
| **Uzbekistan** | **-** | **1** | **-** | **-** | **-** | **-** | **-** |  | **-** | **-** | **-** | **-** | **-** | **-** | **-** | **1** |
| **Yugoslavia** | **-** | **-** | **-** | **-** | **-** | **-** | **-** |  | **2** | **-** | **-** | **-** | **-** | **-** | **-** | **2** |
| **Autochthonous (number)** | **10** | **7** | **10** | **253** | **3** | **10** | **11** | **324** | **513** | **29** | **5** | **366** | **3** | **20** | **116** | **1,680** |
| **Autochthonous (%)** | **100** | **18.4** | **90.9** | **100** | **10.0** | **100** | **91.7** | **89.5** | **61.7** | **100** | **100** | **100** | **100** | **100** | **99.1** | **80.1** |
| **Immigrants**  **(number)** | **-** | **31** | **1** | **-** | **27** | **-** | **1** | **38** | **318** | **-** | **-** | **-** | **-** | **-** | **1** | **417** |
| **Immigrants**  **(%)** | **-** | **81.6** | **9.1** | **-** | **90.0** | **-** | **8.3** | **10.5** | **38.3** | **-** | **-** | **-** | **-** | **-** | **0.9** | **19.9** |
| **Total number** | **10** | **38** | **11** | **253** | **30** | **10** | **12** | **362** | **831** | **29** | **5** | **366** | **3** | **20** | **117** | **2,097** |

**Additional file 1: Table S2**. Clinical management of cysts by stage and location.

| **N. CYSTS** | **N. OBSERVATIONS ASSOCIATED WITH MANAGEMET** | **CYST STAGE** | **ALBENDAZOLE** | **SURGERY + ALBENDAZOLE** | **SURGERY w/o INDICATION OF ASSOCIATED ALBENDAZOLE** | **PERCUTANEOUS TREATMENT + ALBENDAZOLE** | **PERCUTANEOUS TREATMENT w/o INDICATION OF ASSOCIATED ALBENDAZOLE** | **WATCH AND WAIT** |
| --- | --- | --- | --- | --- | --- | --- | --- | --- |
| 604 | 773 | **LIVER** | | | | | | |
|  | 159 | CE1 | 66 | 19 | 35 | 25 | 12 | 2 |
|  | 100 | CE2 | 41 | 21 | 22 | 0 | 15 | 1 |
|  | 94 | CE3a | 41 | 6 | 5 | 11 | 2 | 29 |
|  | 210 | CE3b | 83 | 60 | 4 | 4 | 0 | 59 |
|  | 210 | CE4-CE5 | 17 | 14 | 2 | 0 | 0 | 177 |
| 45 | 46 | **LUNG** | | | | | | |
|  | N/A | | 10 | 19 | 13 | 0 | 0 | 4 |
| 29 | 32 | **SEROSAL CAVITIES (abdomen=16; pelvis=12; thorax=1)** | | | | | | |
|  | 4 | CE1 | 1 | 2 | 1 | 0 | 0 | 0 |
|  | 5 | CE2 | 1 | 2 | 1 | 1 | 0 | 0 |
|  | 13 | CE3b | 7 | 1 | 12 | 0 | 0 | 3 |
|  | 10 | CE4-CE5 | 1 | 0 | 0 | 0 | 0 | 9 |
| 16 | 19 | **SPLEEN** | | | | | | |
|  | 9 | CE1 | 2 | 2 | 1 | 4 | 0 | 0 |
|  | 3 | CE2 | 0 | 2 | 1 | 0 | 0 | 0 |
|  | 2 | CE3a | 0 | 1 | 0 | 0 | 0 | 1 |
|  | 1 | CE3b | 1 | 0 | 0 | 0 | 0 | 0 |
|  | 4 | CE4-CE5 | 2 | 0 | 0 | 0 | 0 | 2 |
| 11 | 26 | **MUSCLE AND SUBCUTANEOUS TISSUE** | | | | | | |
|  | 1 | CE2 | 1 | 0 | 0 | 0 | 0 | 0 |
|  | 18 | CE3b | 13 | 0 | 0 | 0 | 0 | 5 |
|  | 7 | CE4 | 0 | 0 | 0 | 0 | 0 | 7 |
| N/A | 10 | **BONE** | | | | | | |
|  | N/A | | 8 | 2 | 0 | 0 | 0 | 0 |
| 5 | 5 | **KIDNEY** | | | | | | |
|  | 2 | CE1 | 2 | 0 | 0 | 0 | 0 | 0 |
|  | 1 | CE2 | 1 | 0 | 0 | 0 | 0 | 0 |
|  | 1 | CE3b | 0 | 1 | 0 | 0 | 0 | 0 |
|  | 1 | CE4-CE5 | 0 | 0 | 0 | 0 | 0 | 1 |
| 3 | 6 | **PANCREAS** | | | | | | |
|  | 5 | CE3b | 2 | 0 | 0 | 0 | 0 | 3 |
|  | 1 | CE4 | 0 | 0 | 0 | 0 | 0 | 1 |
| 3 | 3 | **HEART** | | | | | | |
|  | 2 | CE2 | 0 | 1 | 1 | 0 | 0 | 0 |
|  | 1 | CE3a | 1 | 0 | 0 | 0 | 0 |  |
